# Calpain activation mediates microgravity-induced myocardial abnormalities in mice via p38 and ERK1/2 MAPK pathways

**DOI:** 10.1074/jbc.RA119.011890

**Published:** 2021-01-13

**Authors:** Liwen Liang, Huili Li, Ting Cao, Lina Qu, Lulu Zhang, Guo-Chang Fan, Peter A. Greer, Jianmin Li, Douglas L. Jones, Tianqing Peng

**Affiliations:** 1 Institutes of Biology and Medical Sciences, Soochow University, Suzhou, China; 2 Department of Pathology and Laboratory Medicine, Western University, London, Ontario, Canada; 3 State Key Laboratory of Space Medicine Fundamentals and Application, China Astronaut Research and Training Center, Beijing, China; 4 Department of Pharmacology and Systems Physiology, University of Cincinnati College of Medicine, Cincinnati, Ohio, USA; 5 Division of Cancer Biology and Genetics, Queen's University Cancer Research Institute, Queen's University, Kingston, Ontario, Canada; 6 Department of Pathology and Molecular Medicine, Queen's University, Kingston, Ontario, Canada; 7 Department of Pathology, First Affiliated Hospital of Wenzhou Medical University, Wenzhou, China; 8 Department of Physiology and Pharmacology, Western University, London, Ontario, Canada; 9 Lawson Health Research Institute of London Health Sciences Centre, London, Ontario, Canada; 10 Department of Medicine, Western University, London, Ontario, Canada

**Keywords:** calpain, ERK1/2, microgravity, myocardial abnormalities, NADPH oxidase, p38, cardiomyocyte, mitogen-activated protein kinase (MAPK), heart

## Abstract

The human cardiovascular system has adapted to function optimally in Earth's 1G gravity, and microgravity conditions cause myocardial abnormalities, including atrophy and dysfunction. However, the underlying mechanisms linking microgravity and cardiac anomalies are incompletely understood. In this study, we investigated whether and how calpain activation promotes myocardial abnormalities under simulated microgravity conditions. Simulated microgravity was induced by tail suspension in mice with cardiomyocyte-specific deletion of *Capns1*, which disrupts activity and stability of calpain-1 and calpain-2, and their WT littermates. Tail suspension time-dependently reduced cardiomyocyte size, heart weight, and myocardial function in WT mice, and these changes were accompanied by calpain activation, NADPH oxidase activation, and oxidative stress in heart tissues. The effects of tail suspension were attenuated by deletion of *Capns1*. Notably, the protective effects of *Capns1* deletion were associated with the prevention of phosphorylation of Ser-345 on p47*^phox^* and attenuation of ERK1/2 and p38 activation in hearts of tail-suspended mice. Using a rotary cell culture system, we simulated microgravity in cultured neonatal mouse cardiomyocytes and observed decreased total protein/DNA ratio and induced calpain activation, phosphorylation of Ser-345 on p47*^phox^*, and activation of ERK1/2 and p38, all of which were prevented by calpain inhibitor-III. Furthermore, inhibition of ERK1/2 or p38 attenuated phosphorylation of Ser-345 on p47*^phox^* in cardiomyocytes under simulated microgravity. This study demonstrates for the first time that calpain promotes NADPH oxidase activation and myocardial abnormalities under microgravity by facilitating p47*^phox^* phosphorylation via ERK1/2 and p38 pathways. Thus, calpain inhibition may be an effective therapeutic approach to reduce microgravity-induced myocardial abnormalities.

Spaceflight or microgravity conditions are associated with myocardial atrophy, which contributes to the functional depression of the heart (1, 2, 3). Depressed myocardial function is an important factor leading to post-flight orthostatic intolerance (4, 5). However, the exact mechanisms that govern the regulation of myocardial atrophy in microgravity are incompletely understood.

Protein degradation systems play important roles in muscle atrophy (6). There are three major protein degradation systems in the cardiovascular system, including the calpain system, autophagy, and the ubiquitin proteasome system (7). Previous studies investigated the muscle RING finger protein-1 (MuRF1), an E3 ubiquitin ligase, and atrogin-1, a striated muscle-specific ubiquitin ligase, in the development of cardiac muscle atrophy under certain conditions (8, 9, 10). It was reported that autophagy is induced during regression of cardiac hypertrophy by unloading of the heart (11, 12) and in myocardial atrophy induced by tail suspension (13), a condition simulating microgravity. Of note, inhibition of autophagy induction reduced myocardial atrophy and dysfunction in tail-suspended rats (13), indicating an important role of autophagy in promoting myocardial atrophy. However, little information is available on the role of calpain in myocardial atrophy under microgravity.

Calpains belong to a family of calcium-dependent proteases with 15 identified isoforms. Two major isoforms of calpain, calpain-1 and calpain-2, are heterodimers consisting of distinct large 80-kDa catalytic subunits encoded by *Capn1* and *Capn2*, respectively, and a common small 28-kDa regulatory subunit encoded by *Capns1*. The regulatory subunit is indispensable for calpain-1 and calpain-2 activities, and thus, deficiency of *Capns1* disrupts calpain-1 and calpain-2 (14). Calpains have been implicated in cardiac injury due to a variety of stressors and the progression of heart failure (15, 16, 17, 18, 19, 20, 21, 22, 23). A previous study demonstrated that mechanical unloading of the heart activated the calpain system and induced myocardial atrophy (24). Interestingly, calpain activation was observed in hearts of rats with tail suspension (25). These prior studies suggest a possible role of calpain in microgravity-induced myocardial atrophy.

NADPH oxidase-derived ROS contributes to muscle atrophy under various conditions (2, 26). In cardiomyocytes, NOX2-containing NADPH oxidase and NOX4 are two predominant isoforms of NADPH oxidase (27, 28). The NOX2-containing NADPH oxidase is composed of a membrane-bound complex (NOX2 or gp91*^phox^* and p22*^phox^*) and a cytosolic complex (p40*^phox^*, p47*^phox^*, p67*^phox^*, and a small G protein, Rac1). Activation of NADPH oxidase initiates multiple steps, mainly including phosphorylation of cytosolic subunits (*e.g.* p47*^phox^*), their translocation to the membranes, and the assembly of both cytosolic and membrane-bound complexes (27, 29). p47*^phox^* has multiple serine phosphorylation sites, among which Ser-345 is located in a mitogen-activated protein kinase (MAPK) consensus sequence that is phosphorylated by p38 and ERK1/2 of the MAPK family (29). Phosphorylation of Ser-345 on p47*^phox^* causes conformational changes of p47*^phox^* by binding the proline isomerase Pin1, thereby facilitating its phosphorylation on other sites, leading to full activation of the NADPH oxidase (29). We recently reported that selective inhibition of NADPH oxidase preserved cardiomyocyte size and heart mass and improved myocardial function in tail-suspended mice (30), underscoring an important role of NADPH oxidase in microgravity-induced myocardial abnormalities. However, it remains unknown how NADPH oxidase activation is modulated in response to microgravity, and it has never been shown whether calpain regulates NADPH oxidase activation under microgravity.

In this study, we investigated whether and how calpain promotes NADPH oxidase activation and oxidative damage, leading to myocardial abnormalities during simulated microgravity.

## Results

### Tail suspension time-dependently induces calpain activation, reduces cardiomyocyte size and heart weight, and promotes myocardial dysfunction in mice

**Figure 1:**
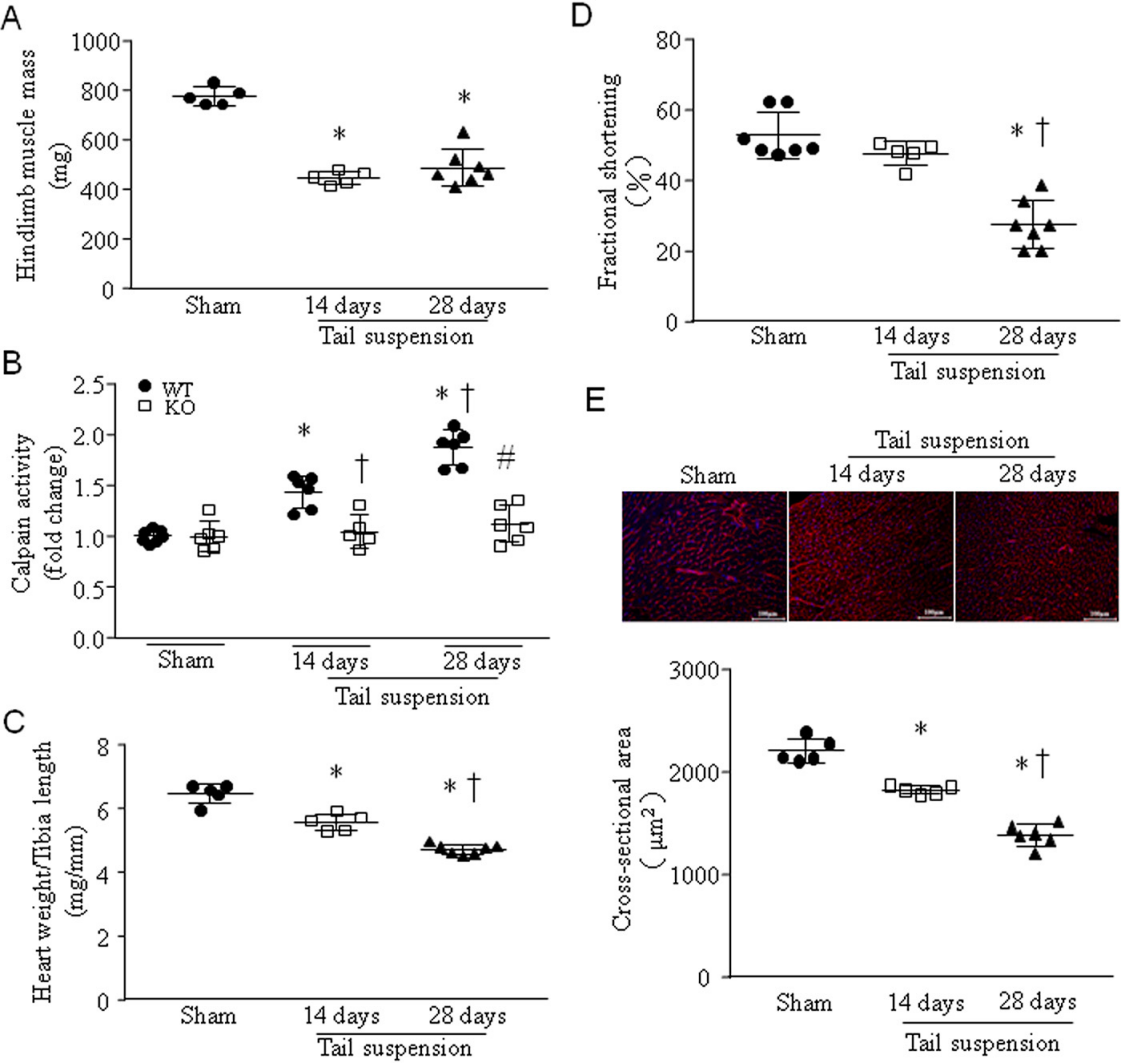
**Tail suspension induces calpain activation, myocardial atrophy, and dysfunction in mice.** Adult male mice were subjected to tail suspension for 14 or 28 days. *A*, hind limb muscle mass. Data are mean ± S.D. (*error bars*), *n* = 5–7 in each group. One-way ANOVA followed by Newman–Keuls test was performed for statistical analysis (*F* = 59.01, *p* < 0.0001). *, *p* < 0.05 *versus* sham. *B*, calpain activities in heart tissues from *Capns1*-KO mice and their WT littermates. Data are mean ± S.D., *n* = 6 in each group. Two-way ANOVA followed by Newman–Keuls test was performed for statistical analysis (interaction, *F* = 19.27, *p* < 0.0001; row factor, *F* = 33.81, *p* < 0.0001; column factor, *F* = 56.78, *p* < 0.0001). *, *p* < 0.05 *versus* sham; †, *p* < 0.05 *versus* 14 days after tail suspension in WT; #, *p* < 0.05 *versus* 28 days after tail suspension in WT. *C*, the ratio of heart weight relative to tibia length. Data are mean ± S.D., *n* = 5–7 in each group. One-way ANOVA followed by Newman–Keuls test was performed for statistical analysis (*F* = 75.13, *p* < 0.0001). *, *p* < 0.05 *versus* sham; †, *p* < 0.05 *versus* 14 days after tail suspension. *D*, fractional shortening (%) was analyzed by echocardiography. Data are mean ± S.D., *n* = 5–7 in each group. One-way ANOVA followed by Newman–Keuls test was performed for statistical analysis (*F* = 32.44, *p* < 0.0001). *, *p* < 0.05 *versus* sham; †, *p* < 0.05 *versus* 14 days after tail suspension. *E*, *top*, a representative staining of wheat germ agglutinin in heart tissue sections; *bottom*, quantitation of cardiomyocyte cross-sectional area. Data are mean ± S.D., *n* = 5–6 in each group. One-way ANOVA followed by Newman–Keuls test was performed for statistical analysis (*F* = 100.8, *p* < 0.0001). *, *p* < 0.05 *versus* sham; †, *p* < 0.05 *versus* 14 days after tail suspension.

Tail suspension for 14 and 28 days significantly resulted in lower hind limb muscle mass, confirming its unloading (Fig. 1*A*). Tail suspension for 14 days resulted in higher levels of calpain activities in WT mouse heart tissues; the levels of calpain activities were even higher in mouse hearts after 28 days of tail suspension (Fig. 1*B*); however, calpain activities were not increased in *Capns1*-KO mouse hearts after 14 and 28 days of tail suspension (Fig. 1*B*). These results indicated calpain activation in tail-suspended mouse hearts. This time-dependent activation of calpain negatively correlated with heart weight (Fig. 1*C* and Table S1), myocardial function (Fig. 1*D* and Table S2), and cardiomyocyte size (Fig. 1*E*) in tail-suspended mice. Tail suspension for 28 days reduced the mitochondrial DNA copies of NADH dehydrogenase subunit 1 (*Mtnd1)* but not ATP production in WT mouse hearts (Fig. S1, A and B).

### Deletion of Capns1 prevents myocardial abnormalities in tail-suspended mice

**Figure 2:**
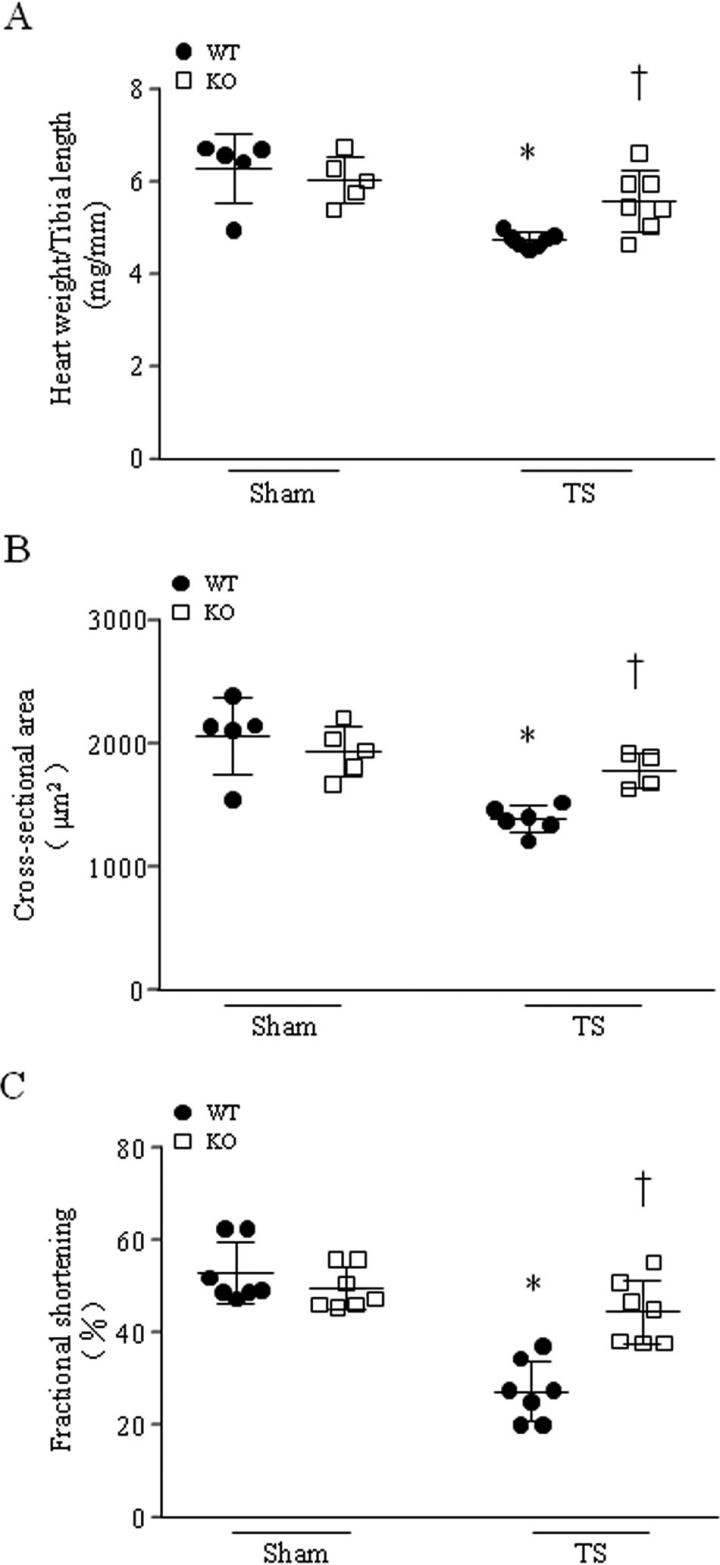
**Tail suspension induces myocardial atrophy and dysfunction in WT mice, which are attenuated in *Capns1*-knockout mice.** Mice with cardiomyocyte-specific deletion of *Capns1* (*KO*) and their WT littermates (*WT*) were subjected to tail suspension (TS) for 28 days. *A*, the heart weight/tibia length ratio was decreased in tail-suspended WT mice. Deletion of *Capns1* prevented the decrease of the heart weight/tibia length ratio. Data are mean ± S.D. (*error bars*), *n* = 5–7 in each group. Two-way ANOVA followed by Newman–Keuls test was performed for statistical analysis (interaction, *F* = 5.749, *p* = 0.0264; row factor, *F* = 19.55, *p* = 0.0003; column factor, *F* = 1.819, *p* = 0.1925). *, *p* < 0.05 *versus* sham + WT; †, *p* < 0.05 *versus* TS + WT. *B*, a 28-day TS resulted in lower cardiomyocyte cross-sectional area in WT mice, which was prevented in KO mice. Data are mean ± S.D., *n* = 4–6 in each group. Two-way ANOVA followed by Newman–Keuls test was performed for statistical analysis (interaction, *F* = 8.137, *p* = 0.011; row factor, *F* = 23.48, *p* = 0.0002; column factor, *F* = 1.929, *p* = 0.1828). *, *p* < 0.05 *versus* sham + WT; †, *p* < 0.05 *versus* TS + WT. *C*, TS resulted in myocardial dysfunction in WT mice as determined by lower fractional shortening (%), which was prevented in KO mice. Data are mean ± S.D., *n* = 7 in each group. Two-way ANOVA followed by Newman–Keuls test was performed for statistical analysis (interaction, *F* = 18.76, *p* = 0.0002; row factor, *F* = 42.35, *p* < 0.0001; column factor, *F* = 8.51, *p* = 0.0075). *, *p* < 0.05 *versus* sham + WT; †, *p* < 0.05 *versus* TS + WT.

28 days after tail suspension, deletion of *Capns1* resulted in greater heart weight (Fig. 2*A*), bigger cardiomyocyte size (Fig. 2*B*), and better myocardial function in *Capns1* mice compared with their WT littermates (Fig. 2*C* and Table S2). Because deletion of *Capns1* has been shown to disrupt the stability and activities of calpain-1 and calpain-2 in the heart (23), these results demonstrate that inhibition of calpain prevents myocardial abnormalities under microgravity. However, cardiomyocyte-specific disruption of calpain did not prevent hind limb muscle mass loss in tail-suspended mice (Table S1).

### Deficiency of Capns1 inhibits NADPH oxidase activation and oxidative stress in tail-suspended mouse hearts

**Figure 3:**
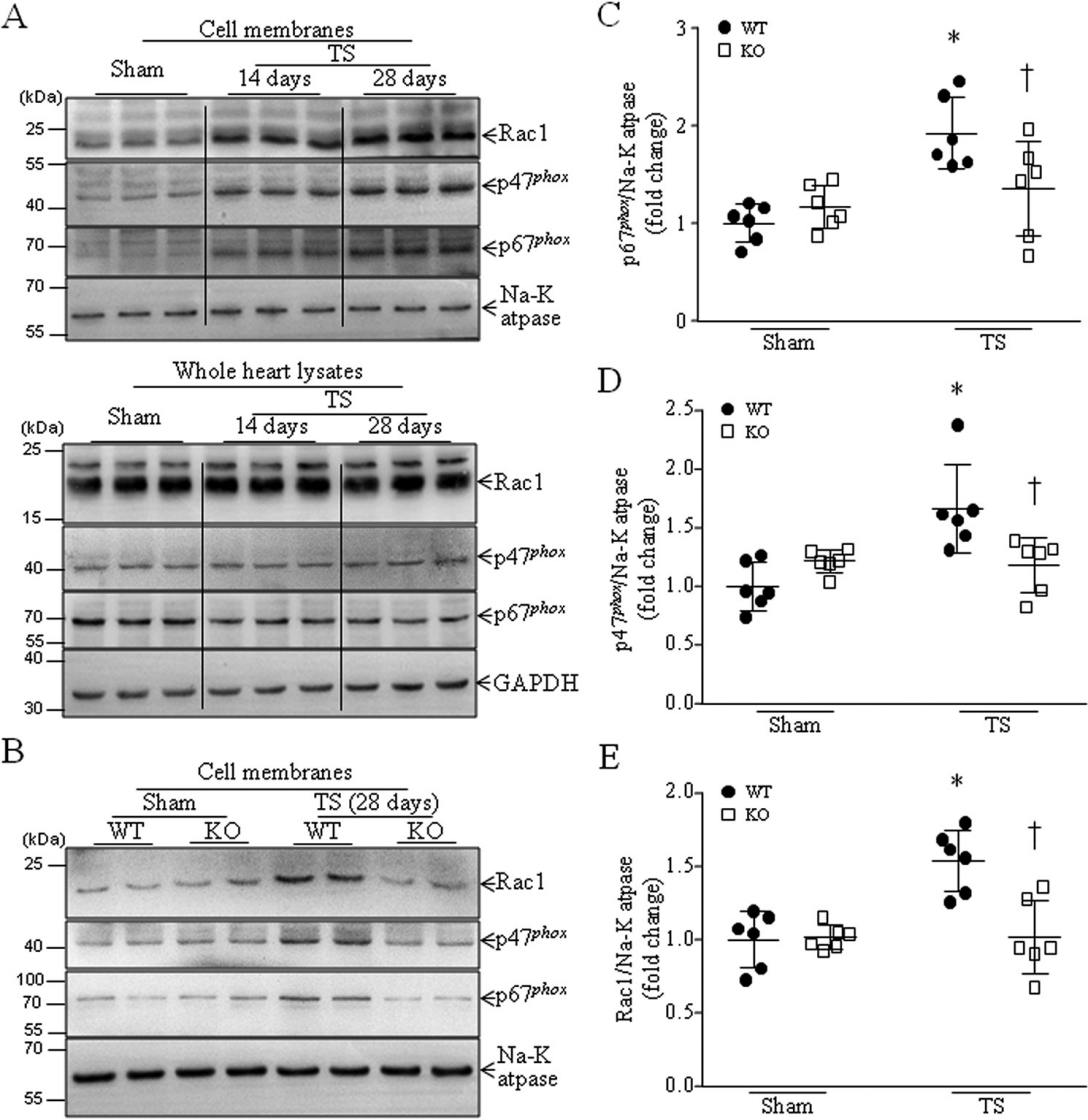
**Tail suspension induces NADPH oxidase activation in WT mice, which is prevented in *Capns1*-knockout mice.** Mice with cardiomyocyte-specific deletion of *Capns1* (*KO*) and their WT littermates (*WT*) were subjected to TS for 14 or 28 days. Cell membranes were isolated from mouse hearts, and NADPH oxidase activation was determined by measuring p47*^phox^*, p67*^phox^*, and Rac1 in cell membrane fractions relative to Na^+^/K^+^-ATPase. *A*, representative Western blots from three different hearts for p47*^phox^*, p67*^phox^*, and Rac1 in cell membranes (*top*) and whole heart lysates (*bottom*). *B*, a representative Western blot from two of six different hearts for p47*^phox^*, p67*^phox^*, Rac1, and Na^+^/K^+^-ATPase in the membranes. *C–E*, quantitation for p67*^phox^* (*C*), p47*^phox^* (*D*), and Rac1 (*E*) relative to Na^+^/K^+^-ATPase. Data are mean ± S.D. (*error bars*), *n* = 6 in each group. Two-way ANOVA followed by Newman–Keuls test was performed for statistical analysis. *C*, interaction, *F* = 22.28, *p* < 0.0001; row factor, *F* = 3.988, *p* = 0.052; column factor, *F* = 2.522, *p* = 0.1194. *D*, interaction, *F* = 12.62, *p* = 0.002; row factor, *F* = 6.15, *p* = 0.0222; column factor, *F* = 1.455, *p* = 0.2417. *E*, interaction, *F* = 6.97, *p* = 0.0134; row factor, *F* = 1.898, *p* = 0.1792; column factor, *F* = 7.08, *p* = 0.0128. *, *p* < 0.05 *versus* sham + WT; †, *p* < 0.05 *versus* TS + WT.

Our recent study reported that simulated microgravity induced NADPH oxidase activation in tail-suspended mouse hearts (30). As shown in Fig. 3*A*, tail suspension for 14 days resulted in significantly higher protein levels of cytosolic subunits of NADPH oxidase, including Rac1, p47*^phox^*, and p67*^phox^* in cell membranes, indicative of NADPH oxidase activation, as activation of NADPH oxidase requires the translocation of its cytosolic subunits to the membranes (29), and their levels in cell membranes further increased 28 days after tail suspension, whereas their levels in whole heart lysates were not altered. The activation of NADPH oxidase correlated with calpain activation in tail-suspended mouse hearts in a time-dependent manner (Fig. 1*B*). Notably, deficiency of *Capns1* reduced the protein levels of Rac1, p47*^phox^*, and p67*^phox^* in the cell membranes of tail-suspended mouse hearts, indicating that disruption of calpain-1 and calpain-2 prevents NADPH oxidase activation in tail-suspended mouse hearts (Fig. 3, *B–E*).

**Figure 4:**
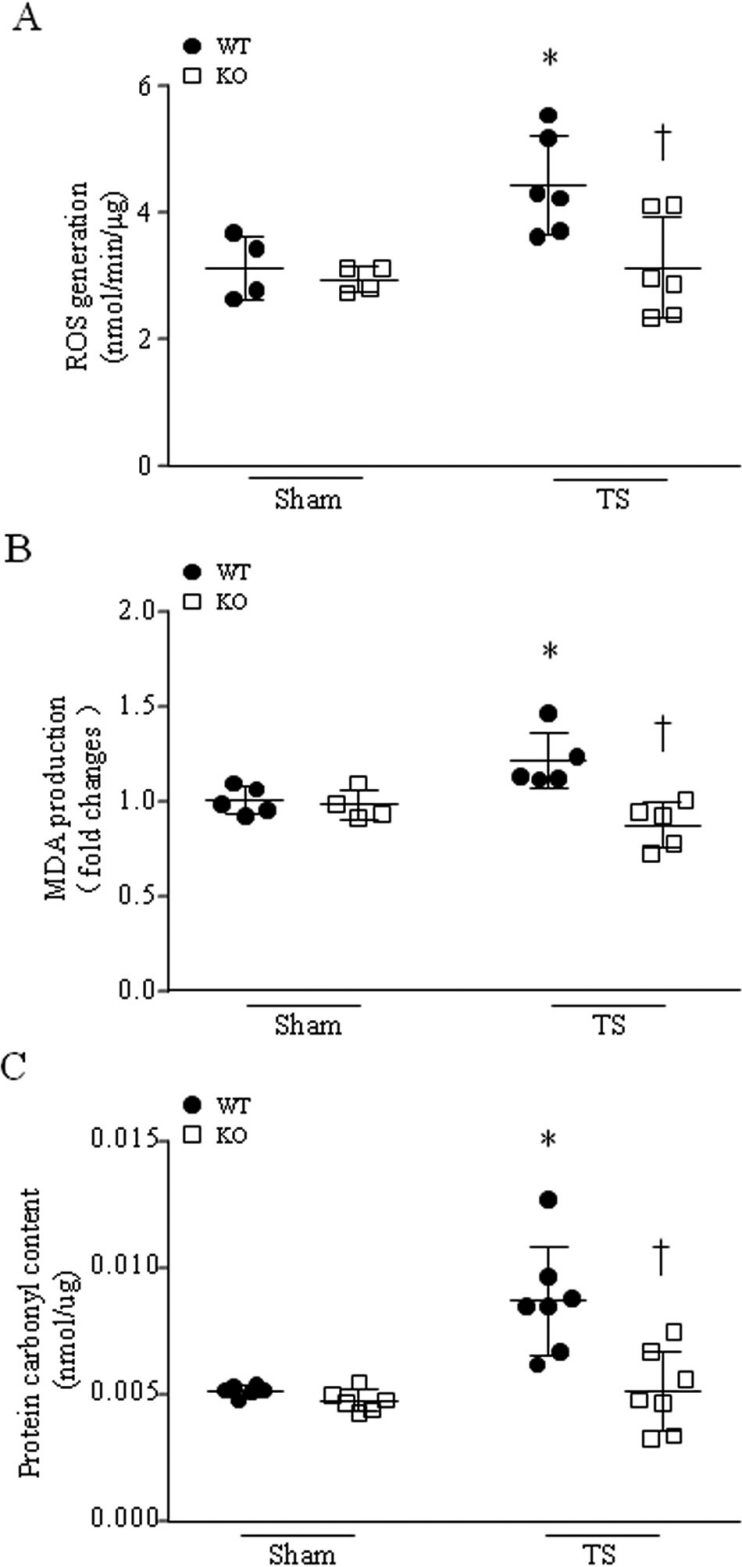
**Deletion of *Capns1* inhibits tail suspension-induced oxidative stress in mouse hearts.** Mice with cardiomyocyte-specific deletion of *Capns1* (*KO*) and their WT littermates (*WT*) were subjected to TS for 28 days. Oxidative stress was assessed by measuring ROS production (*A*), MDA production (*B*), and protein carbonyl content (*C*) in mouse hearts. Data are mean ± S.D. (*error bars*), *n* = 4–7 in each group. Two-way ANOVA followed by Newman–Keuls test was performed for statistical analysis. *A*, interaction, *F* = 3.358, *p* = 0.0855; row factor, *F* = 5.995, *p* = 0.0263; column factor, *F* = 5.915, *p* = 0.0271. *B*, interaction, *F* = 9.633, *p* = 0.0073; row factor, *F* = 1.004, *p* = 0.3322; column factor, *F* = 12.56, *p* = 0.0029. *C*, interaction, *F* = 7.275, *p* = 0.0194; row factor, *F* = 2.682, *p* = 0.1274; column factor, *F* = 9.306, *p* = 0.0101. *, *p* < 0.05 *versus* sham + WT; †, *p* < 0.05 *versus* TS + WT.

In line with activation of NADPH oxidase, more ROS production was observed in heart tissues of tail-suspended mice compared with sham animals (Fig. 4*A*), as was greater protein carbonyl content and malondialdehyde (MDA) production, indicators of oxidized protein and lipid peroxidation, respectively (Fig. 4, *B* and *C*). However, ROS production was much less in tail-suspended *Capns1*-KO mice compared with their WT littermates (Fig. 4*A*). Similarly, deficiency of *Capns1* also resulted in less oxidative damage in hearts of tail-suspended mice as determined by less protein carbonyl and MDA contents (Fig. 4, *B* and *C*).

Because cardiomyocytes predominantly express NOX2 and NOX4, we also analyzed NOX4 expression, as NOX4 is primarily regulated through transcriptional mechanisms (31). Tail suspension resulted in higher protein levels of NOX4 in tail-suspended mouse hearts (Fig. S2). However, deficiency of *Capns1* did not change the higher protein levels of NOX4 due to tail suspension (Fig. S2). In addition, neither tail suspension nor deletion of *Capns1* changed mitochondrial ROS generation and xanthine oxidase activity in tail-suspended mouse hearts (Fig. S3 (A–C)).

Increased ROS production and consequent oxidative damage may also result from a defect of antioxidant systems. Therefore, we measured the main antioxidant enzymes in hearts. As shown in Fig. S4 (A–C), neither tail suspension nor deletion of C*apns1* changed the activities of SOD, GPx, and catalase in heart tissues. These results exclude the possibility that changes of antioxidant enzymes play a role in calpain activation-associated oxidative stress in tail-suspended mouse hearts.

### Calpain deficiency is associated with reduced phosphorylation of Ser-345 on p47^phox^ in tail-suspended mouse hearts and cultured cardiomyocytes under microgravity

**Figure 5:**
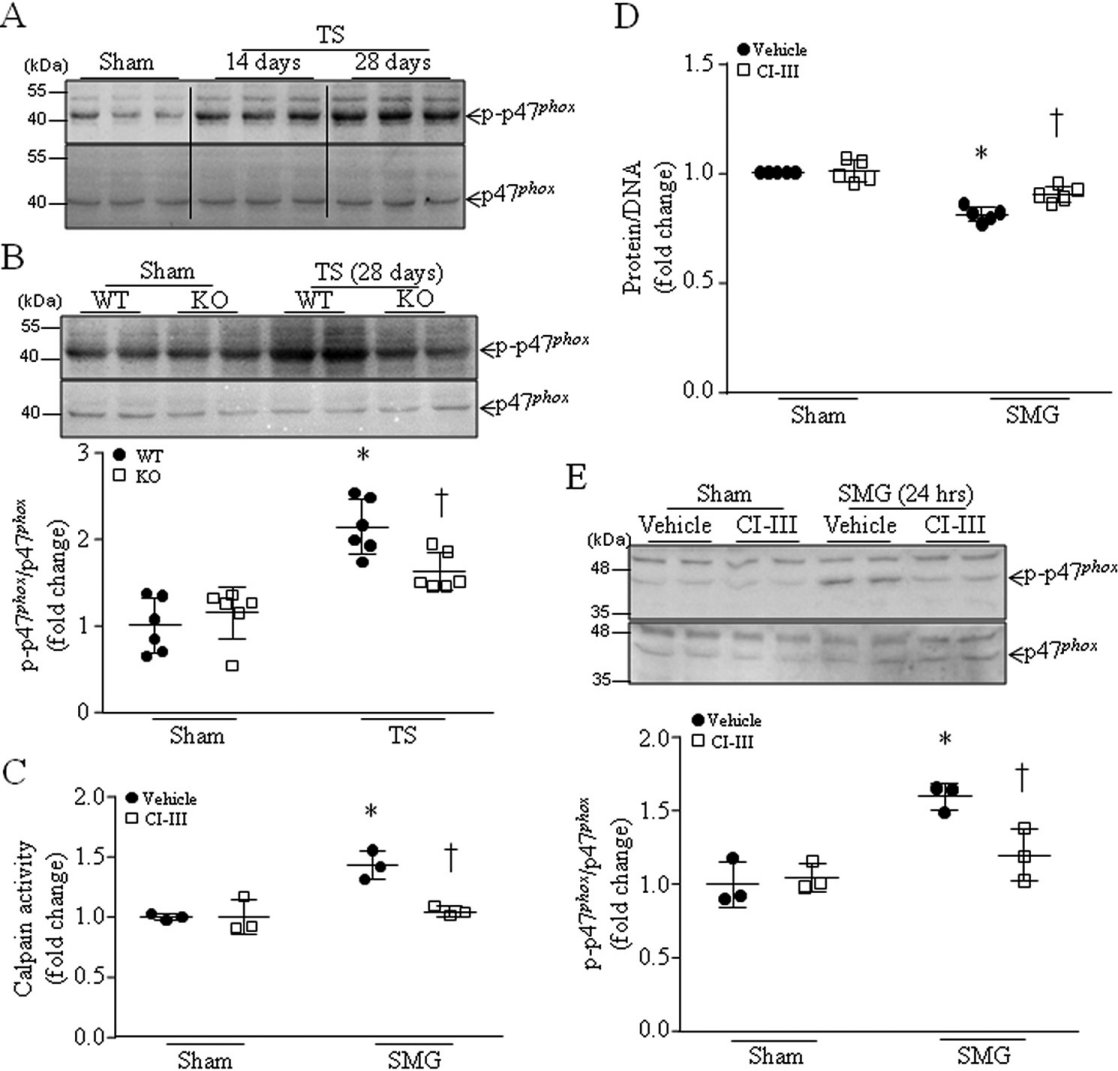
**Inhibition of calpain prevents phosphorylation of Ser-345 on p47*^phox^* in tail-suspended mouse hearts and cultured cardiomyocytes in response to simulated microgravity.***A* and *B*, mice with cardiomyocyte-specific deletion of *Capns1* (*KO*) and their WT littermates (*WT*) were subjected to TS for 14 or 28 days. *A*, a representative Western blot from three different hearts in each group for phosphorylated p47*^phox^* (Ser-345) and total p47 *^phox^*. *B*, *top*, a representative Western blot from two of six different hearts in each group for phosphorylated p47*^phox^* (Ser-345) and total p47 *^phox^*; *bottom*, quantitation for phosphorylated p47*^phox^* (Ser-345) relative to total p47 *^phox^*. Data are mean ± S.D. (*error bars*), *n* = 6 in each group. Two-way ANOVA followed by Newman–Keuls test was performed for statistical analysis (interaction, *F* = 14.2, *p* = 0.0008; row factor, *F* = 27.2, *p* < 0.0001; column factor, *F* = 9.388, *p* = 0.0048). *, *p* < 0.05 *versus* sham + WT or sham + KO. †, *p* < 0.05 *versus* TS + WT or sham + KO. *C–E*, neonatal mouse cardiomyocytes were subjected to a condition of simulated microgravity (SMG) in the presence of calpain inhibitor-III (*CI-III*) or vehicle for 24 h. *C*, calpain activities. *D*, total protein/DNA ratio in cardiomyocyte lysates. *E*, *top*, a representative Western blot from three different cell cultures with each in duplicate for phosphorylated p47*^phox^* (Ser-345) relative to total p47*^phox^*; *bottom*, quantitation for phosphorylated p47*^phox^* (Ser-345) relative to total p47*^phox^*. Data are mean ± S.D., *n* = 3–5 in each group. Two-way ANOVA followed by Newman–Keuls test was performed for statistical analysis. *C*, interaction, *F* = 11.75, *p* = 0.0090; row factor, *F* = 18.26, *p* = 0.0027; column factor, *F* = 12.13, *p* = 0.0083. *D*, interaction, *F* = 7.366, *p* = 0.0153; row factor, *F* = 86.86, *p* < 0.0001; column factor, *F* = 9.917, *p* = 0.0062. *E*, interaction, *F* = 5.361, *p* = 0.0313; row factor, *F* = 31.8, *p* < 0.0001; column factor, *F* = 5.368, *p* = 0.0312. *, *p* < 0.05 *versus* sham + vehicle; †, *p* < 0.05 *versus* SMG + vehicle.

To investigate the potential mechanisms by which calpain promoted NADPH oxidase activation, we focused on the phosphorylation state of p47*^phox^* as a readout for translocation of this cytosolic subunit of NADPH oxidase to the cell membranes (29). Similar to the translocation of NADPH oxidase cytosolic subunits to the membranes, the levels of phosphorylation of Ser-345 on p47*^phox^* were higher in mouse hearts 14 and 28 days after tail suspension (Fig. 5*A*). Interestingly, in tail-suspended mice, deletion of *Capns1* resulted in lower levels of phosphorylation of Ser-345 on p47*^phox^* (Fig. 5*B*), suggesting that calpain promotes phosphorylation of Ser-345 on p47*^phox^* in mediating NADPH oxidase activation in tail-suspended mouse hearts.

To provide further evidence supporting the role of calpain in promoting p47*^phox^* phosphorylation, we simulated microgravity in cultured cardiomyocytes using the rotary cell culture system. Simulated microgravity increased calpain activities (Fig. 5*C*), and this was correlated with a reduction of total protein/DNA ratio (Fig. 5*D*) and an increase in phosphorylation of Ser-345 on p47*^phox^* (Fig. 5*E*). Incubation with calpain inhibitor-III prevented calpain activation, increased total protein/DNA ratio, and attenuated microgravity-induced phosphorylation of Ser-345 on p47*^phox^* in cardiomyocytes (Fig. 5, *C–E*). These results recapitulated the inhibitory effect of calpain disruption on p47*^phox^* phosphorylation in tail-suspended mouse hearts.

### MAPK signaling mediates calpain-promoted phosphorylation of Ser-345 on p47^phox^ during microgravity

**Figure 6:**
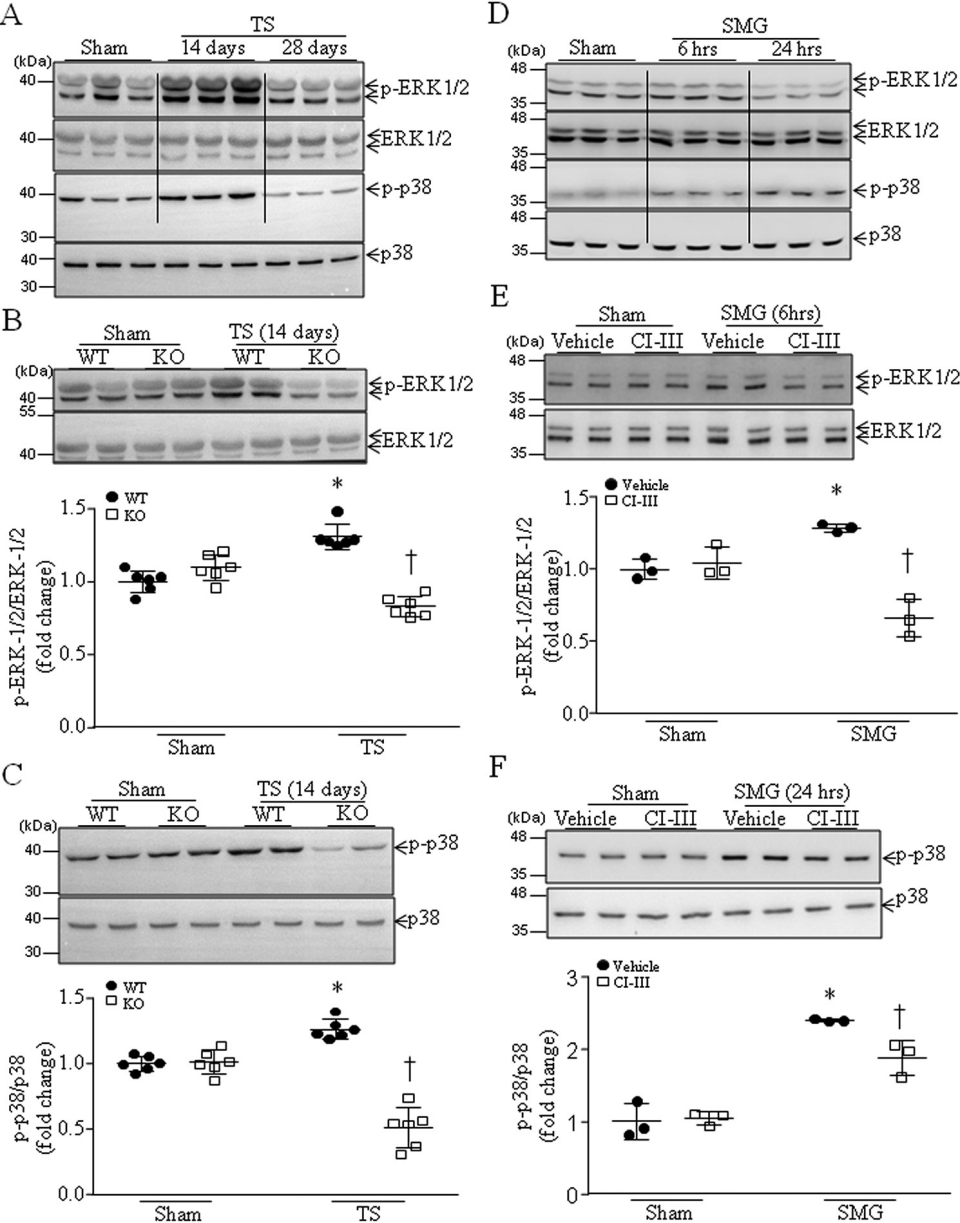
**Tail suspension induces activation of p38 and ERK1/2 in WT mice, which is attenuated in *Capns1*-knockout mice, and inhibition of calpain attenuates phosphorylation of ERK1/2 and p38 in cardiomyocytes under microgravity.***A–C*, mice with cardiomyocyte-specific deletion of *Capns1* (*KO*) and their WT littermates (*WT*) were subjected to tail suspension (*TS*) for 14 or 28 days. *A*, a representative Western blot from three different hearts in each group for total and phosphorylated p38 and ERK1/2. *B*, *top*, a representative Western blot from two of six different hearts in each group for total and phosphorylated ERK1/2; *bottom*, quantitation for phosphorylated ERK1/2 (*p-ERK1/2*) relative to total ERK1/2. *C*, *top*, a representative Western blot from two of six different hearts in each group for total and phosphorylated p38; *bottom*, quantitation for phosphorylated p38 (*p-p38*) relative to total p38. Data are mean ± S.D. (*error bars*), *n* = 6 in each group. Two-way ANOVA followed by Newman–Keuls test was performed for statistical analysis. *B*, interaction, *F* = 39.53, *p* < 0.0001; row factor, *F* = 0.1185, *p* = 0.7366; column factor, *F* = 19.48, *p* = 0.0008. *C*, interaction, *F* = 82.32, *p* < 0.0001; row factor, *F* = 14.01, *p* = 0.0028; column factor, *F* = 101.8, *p* < 0.0001. *, *p* < 0.05 *versus* sham + WT; †, *p* < 0.05 *versus* KO + vehicle. *D–F*, neonatal mouse cardiomyocytes were subjected to SMG in the presence of calpain inhibitor-III or vehicle for 6 and 24 h. *D*, a representative Western blot from three different cell cultures for total and phosphorylated p38 and ERK1/2. *E*, *top*, a representative Western blot from three different cell cultures with each in duplicate for total and phosphorylated ERK1/2; *bottom*, quantitation for phosphorylated ERK1/2 (p-ERK1/2) relative to total ERK1/2. *F*, *top*, a representative Western blot from three different cell cultures with each in duplicate for total and phosphorylated p38; *bottom*, quantitation for phosphorylated p38 (*p-p38*) relative to total p38. Data are mean ± S.D., *n* = 3 in each group. Two-way ANOVA followed by Newman–Keuls test was performed for statistical analysis. *E*, interaction, *F* = 46.37, *p* < 0.0001; row factor, *F* = 5.254, *p* = 0.0329; column factor, *F* = 42.27, *p* < 0.0001. *F*, interaction, *F* = 7.9, *p* = 0.0108; row factor, *F* = 155.2, *p* < 0.0001; column factor, *F* = 5.059, *p* = 0.0359. *, *p* < 0.05 *versus* sham + vehicle; †, *p* < 0.05 *versus* SMG + vehicle.

Because phosphorylation of Ser-345 on p47*^phox^* is mediated by ERK1/2 and p38 (29), we analyzed the phosphorylation of ERK1/2 and p38 in tail-suspended mouse hearts. As shown in Fig. 6*A*, the levels of phosphorylated ERK1/2 and p38 were higher in WT mouse hearts 14 days after tail suspension than those of the sham group, and 28 days after tail suspension they were lower than those of the sham group. Thus, we analyzed phosphorylated ERK1/2 and p38 in *Capns1*-KO mice and their WT littermates 14 days after tail suspension. As shown in Fig. 6 (*B* and *C*), the levels of phosphorylated ERK1/2 and p38 were much lower in *Capns1*-KO mice compared with their WT littermates 14 days after tail suspension. These results suggest that calpain may promote phosphorylation of ERK1/2 and p38 primarily in the first 2 weeks in tail-suspended mouse hearts.

To further address this in cultured cardiomyocytes, we showed that the protein levels of phosphorylated ERK1/2 were higher 6 h but not 24 h after simulated microgravity (Fig. 6*D*). Incubation with calpain inhibitor-III prevented ERK1/2 phosphorylation in cardiomyocytes 6 h after simulated microgravity (Fig. 6*E*).

The protein levels of phosphorylated p38 were higher 6 h and even higher 24 h after simulated microgravity (Fig. 6*D*). We then chose to determine the effect of calpain inhibition on p38 phosphorylation 24 h after simulated microgravity. Similarly, incubation with calpain inhibitor-III prevented p38 phosphorylation (Fig. 6*F*).

**Figure 7:**
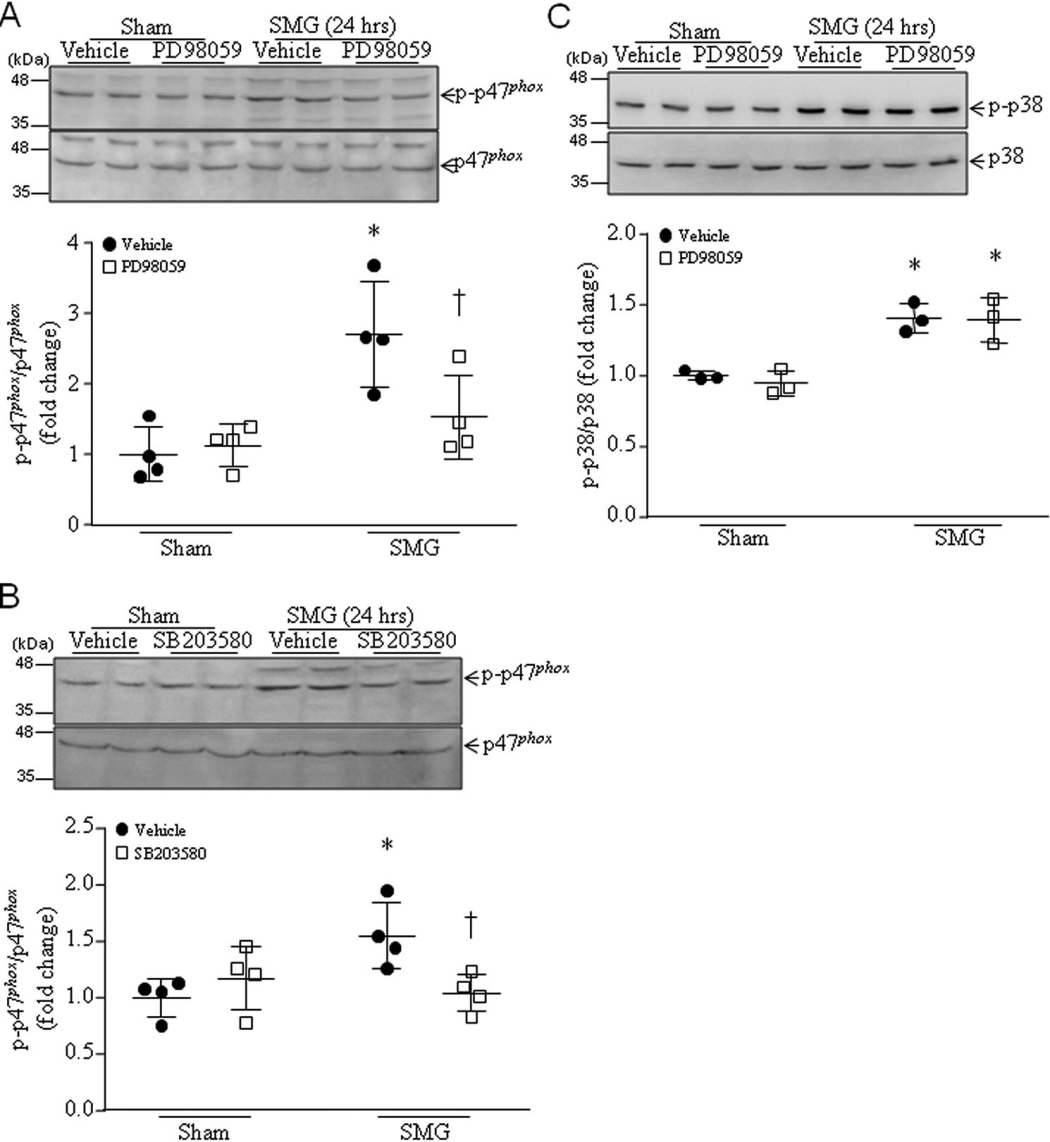
**Inhibition of ERK1/2 and p38 prevents phosphorylation of Ser-345 on p47*^phox^* in cultured cardiomyocytes in response to simulated microgravity.** Neonatal mouse cardiomyocytes were subjected to SMG in the presence of PD98059, SB203580, or vehicle for 24 h. *A* and *B*, *top*, a representative Western blot from three different cell cultures with each in duplicate for total and phosphorylated p47*^phox^* (Ser-345); *Bottom*, quantitation for phosphorylated p47*^phox^* relative to total p47*^phox^*. *C*, effect of ERK1/2 inhibition on p38 phosphorylation in cardiomyocytes after SMG. *Top*, a representative Western blot from three different cell cultures with each in duplicate for total and phosphorylated p38; *bottom*, quantitation for phosphorylated p38 (*p-p38*) relative to total p38. Data are mean ± S.D. (*error bars*), *n* = 3 in each group. Two-way ANOVA followed by Newman–Keuls test was performed for statistical analysis. *A*, interaction, *F* = 5.958, *p* = 0.0311; row factor, *F* = 15.48, *p* = 0.002; column factor, *F* = 3.788, *p* = 0.0754. *B*, interaction, *F* = 8.13, *p* = 0.0145; row factor, *F* = 3.085, *p* = 0.1045; column factor, *F* = 2.035, *p* = 0.1792. *C*, interaction, *F* = 0.1223, *p* = 0.7356; row factor, *F* = 48.81, *p* = 0.0001; column factor, *F* = 0.29, *p* = 0.6049. *, *p* < 0.05 *versus* sham + vehicle; †, *p* < 0.05 *versus* SMG + vehicle.

Importantly, blockage of ERK1/2 or p38 activation with PD98059 or SB203580, respectively, attenuated phosphorylation of Ser-345 on p47*^phox^* in cultured cardiomyocytes after 24 h of simulated microgravity (Fig. 7, *A* and *B*). Because the time-course analysis for ERK1/2 and p38 phosphorylation showed that ERK1/2 phosphorylation reached the highest levels at 6 h, whereas p38 phosphorylation was at the highest level in cardiomyocytes at 24 h after simulated microgravity (Fig. 6*B*), we therefore determined whether ERK1/2 mediated p38 phosphorylation induced by microgravity. However, inhibition of ERK1/2 with PD98059 did not affect the phosphorylated levels of p38 in cardiomyocytes following simulated microgravity (Fig. 7*C*), suggesting that ERK1/2 and p38 independently facilitate p47*^phox^* phosphorylation in microgravity, whereas ERK1/2 and p38 exert their roles at early and relatively late phases of microgravity, respectively. In support of this conclusion, co-incubation of PD98059 and SB20358 did not further inhibit p47*^phox^* phosphorylation in microgravity compared with PD98059 or SB203580 alone (Fig. S5). Taken together, these results suggest that calpain activation mediates microgravity-induced phosphorylation of Ser-345 on p47*^phox^* through p38 and ERK1/2 signaling in cardiomyocytes.

### Inhibition of calpain does not affect MuRF1 and atrogin-1 expression in cultured cardiomyocytes under microgravity

Because both MuRF1 (8, 9, 10) and atrogin-1 (32, 33) have been implicated in promoting cardiac muscle atrophy or inhibiting cardiac hypertrophy, we also analyzed the protein levels of MuRF1 and atrogin-1 in cardiomyocytes. Simulated microgravity resulted in higher levels of MuRF1 and atrogin-1 in cultured cardiomyocytes; however, inhibition of calpain did not change the protein levels of MuRF1 and atrogin-1 in cardiomyocytes under normal conditions or microgravity (Fig. S6).

## Discussion

The major findings of this study are that disruption of calpain preserves cardiomyocyte size, heart mass, and myocardial function in tail-suspended mice, indicating an important role of calpain in myocardial atrophy under microgravity. Furthermore, calpain activation mediates NADPH oxidase activation by facilitating p38 and ERK1/2 activation and subsequent phosphorylation of Ser-345 on p47*^phox^* in cardiomyocytes in response to microgravity. Thus, this study also reveals an unrecognized role of calpain in promoting NADPH oxidase activation via p38 and ERK1/2 signaling during microgravity.

Calpain activation was reported in heart tissues from tail-suspended rats (25) and unloading mouse and human hearts (24). In line with these prior observations, this study demonstrates that tail suspension induces calpain activation in mouse hearts. Importantly, deletion of *Capns1* preserved cardiomyocyte size and heart mass and protected myocardial function in tail-suspended mice, underscoring a critical role of calpain in microgravity-induced myocardial atrophy and dysfunction. Our *in vitro* study using cultured cardiomyocytes further supports the role of calpain in myocardial atrophy, as inhibition of calpain prevented the reduction of protein/DNA ratio after simulated microgravity. Although a previous study reported that transgenic overexpression of calpastatin failed to reduce myocardial atrophy in a mouse model of heart transplantation (24), the transplanted heart does not have any loading of its left ventricular chamber, a condition totally different from the heart of a tail-suspended mouse. As further evidence in support of our finding, inhibition of calpain by transgenic overexpression of calpastatin slowed muscle waste during murine muscle disuse (34). Additionally, there is usually a compensatory increase in catecholamine release after returning from a long-term spaceflight (25). We have reported that incubation with the catecholamine, norepinephrine, induces calpain activation and apoptosis in cardiomyocytes (35). Along with this, a prior study showed that calpain activation was induced and contributed to myocardial apoptosis in rats during the recovery period after tail suspension (25). Thus, calpain may represent a useful target for prevention and therapy for myocardial disorders during and after microgravity.

Our recent study demonstrated that NADPH oxidase is activated and oxidative stress is induced in tail-suspended mouse hearts and that inhibition of NADPH oxidase with apocynin preserves cardiomyocyte size and heart weight and improves myocardial function in tail-suspended mice (30). This finding suggests an important role of NADPH oxidase in myocardial abnormalities under microgravity. Although activation of NADPH oxidase has been implicated in a variety of cardiac diseases (36) and modulation of NADPH oxidase activation has received intensive attention, the molecular mechanisms underlying NADPH oxidase activation remain poorly understood. An important finding of this study is that calpain activation promotes NADPH oxidase activation in tail-suspended mouse hearts. This was demonstrated by determining the translocation of its cytosolic subunits, including p47*^phox^*, p67*^phox^*, and Rac1, to the cell membranes and concomitant oxidative stress. Our study provides further evidence suggesting that calpain activation promotes phosphorylation of Ser-345 on p47*^phox^* in tail-suspended mouse hearts. Studies have implicated ERK1/2 and p38 in mediating phosphorylation of Ser-345 on p47*^phox^* (29). In line with this, we report that the protein levels of phosphorylated ERK1/2 and p38 are higher in cultured cardiomyocytes under microgravity and in tail-suspended mouse hearts. Of note, deletion of *Capns1* or inhibition of calpain prevented the difference in phosphorylated ERK1/2 and p38 in tail-suspended mouse hearts or cultured cardiomyocytes under microgravity, respectively. Furthermore, pharmacological inhibition of ERK1/2 and p38 attenuated phosphorylation of Ser-345 on p47*^phox^* in cultured cardiomyocytes under microgravity. Thus, we argue that deletion of calpain inhibits phosphorylation of Ser-345 on p47*^phox^* by blocking ERK1/2 and p38 activation, thereby preventing translocation of cytosolic subunits of NADPH oxidase to the membrane, NADPH oxidase activation, and oxidative stress in tail-suspended mouse hearts. Nevertheless, future studies are needed to determine how calpain mediates ERK1/2 and p38 activation during microgravity. It is important to mention that simulated microgravity induces ERK1/2 and p38 activation 14 days but not 28 days after tail suspension, suggesting that ERK1/2 and p38 activation accounts for NADPH oxidase at the early phase of microgravity. This is indeed supported by *in vitro* studies using cultured cardiomyocytes. Moreover, inhibition of ERK1/2 or p38 attenuated but did not completely block p47*^phox^* phosphorylation in cardiomyocytes induced by simulated microgravity. It is therefore possible that additional mechanisms mediate NADPH oxidase activation and subsequent cardiomyocyte atrophy during the late phase of microgravity, which merits further investigation.

Interestingly, our data do not support any role of calpain in NOX4 expression in cultured cardiomyocytes and mouse hearts under microgravity. In addition to NOX2-containing NADPH oxidase and NOX4, other main sources of ROS generation are proposed in cardiomyocytes, including mitochondria and xanthine oxidase (37, 38, 39). However, deletion of *Capns1* did not change mitochondrial ROS generation and xanthine oxidase activity during microgravity in tail-suspended mouse hearts. It is important to mention that excessive ROS production and subsequent oxidative stress is the consequence of an imbalance between ROS generation and antioxidant mechanisms (40, 41). By examining the main antioxidant enzymes, including SOD, catalase, and GPx, this study demonstrated that deletion of *Capns1* had no impact on the activities of these antioxidant enzymes and, thus, rules out the possibility that defects of the main antioxidant enzymes contributed to calpain-mediated oxidative stress in tail-suspended mouse hearts.

In addition to calpain, autophagy and the ubiquitin proteasome system have been implicated in protein degradation in the cardiovascular system (7), which contributes to muscle atrophy. Indeed, the ubiquitin proteasome system has been reported to promote cardiac muscle atrophy. For example, both MuRF1 and atrogin-1 expression are implicated in promoting cardiac muscle atrophy or inhibiting cardiac muscle hypertrophy, and inhibition of either reduces cardiac muscle atrophy under certain conditions (8, 9, 10, 32, 33). In the present study, we also report that simulated microgravity induces an increase in MuRF1 and atrogin-1 protein levels in cultured cardiomyocytes. However, inhibition of calpain did not attenuate the higher protein levels of MuRF1 and atrogin-1 in cardiomyocytes under microgravity. Considering that calpain mediates NADPH oxidase activation and our recent study demonstrates that inhibition of NADPH oxidase did not affect MuRF1 expression in tail-suspended mouse hearts (30), the calpain/NADPH oxidase pathway may represent a new mechanism contributing to microgravity-induced myocardial atrophy, which is independent of MuRF-1 and atrogin-1.

Although myocardial atrophy weakens myocardial contractility, the underlying mechanisms remain to be determined. Our data show that tail suspension for 14 and 28 days reduced mitochondrial *Mtnd1* DNA copies in mouse hearts. However, ATP production remains unchanged in heart tissues after tail suspension. This disparity between mitochondrial DNA copies and ATP production may be due to a reduction in cardiomyocyte size after tail suspension (by about 33% in Fig. 1*E*) as a smaller cardiomyocyte may have a lesser number of mitochondria, whereas ATP production remains the same per unit of cell volume. Thus, a reduction of mitochondrial DNA copies is not a potential mechanism for myocardial dysfunction induced by microgravity. Future studies are needed to determine the detailed mechanisms by which microgravity induces myocardial dysfunction.

In summary, we have demonstrated an important role of calpain in promoting myocardial abnormalities in tail-suspended mice. Thus, targeting calpain may be a useful strategy to protect the heart under conditions of microgravity. Given that pharmacological inhibitors of calpain are under clinical trials, our findings provide important preclinical evidence to support future translational studies.

## Experimental procedures

### Animals

This investigation conforms to the Guide for the Care and Use of Laboratory Animals published by the United States National Institutes of Health (8th Edition, 2011). Breeding pairs of C57BL/6 mice were purchased from the Jackson Laboratory. Mice with cardiomyocyte-specific disruption of *Capns1* (*Capns1*-KO) were generated by breeding mice bearing the targeted *Capns1^PZ^* allele containing *loxP* sites flanking essential coding exons and mice with cardiomyocyte-specific expression of Cre recombinase under the control of α-myosin heavy chain as we described recently (23). Deletion of *Capns1* was confirmed by analyzing *Capns1* mRNA expression in *Capns1*-KO mouse hearts as described previously (23) (Fig. S7). All mice used in this study, including controls, were littermates of the same generation. A breeding program was implemented at Soochow University's animal care facilities. All experimental protocols were approved by the Animal Use Subcommittee at Soochow University (Suzhou, China).

### Experimental protocol

Tail suspension was performed in *Capns1*-KO mice and their WT littermates (males aged 2 months) to simulate microgravity using the methods described previously (30, 42). Briefly, a piece of tape was attached to both the tail and a swivel tied to a horizontal bar at the top of cage. Mice had free access to food and water during the tail suspension. 14 or 28 days later, mice were subjected to various experiments.

### Echocardiography

Animals were lightly anesthetized with inhalant isoflurane (0.5–1%) and imaged using a 40-MHz linear array transducer attached to a preclinical ultrasound system (Vevo 2100, FUJIFILM VisualSonics, Toronto, Canada) with nominal in-plane spatial resolution of 40 μm (axial) × 80 μm (lateral). M-mode and two-dimensional parasternal short-axis scans (133 frames/s) at the level of the papillary muscles were used to assess changes in left ventricular (LV) end-systolic inner diameter, LV end-diastolic inner diameter, LV posterior wall thickness in end-diastole and end-systole, fractional shortening (%), and ejection fraction.

### Histological analyses

Mice were euthanized by cervical dislocation under anesthesia using a mixture of ketamine (100 mg/kg)/xylazine (5 mg/kg, intraperitoneally). Heart tissues were collected, fixed, processed, and sectioned. For cardiomyocyte size measurement, several cross-sections of the whole heart (5 μm thick) were prepared and stained for membranes and nuclei with FITC-conjugated wheat germ agglutinin (Thermo Fisher Scientific) and Hoechst 33342 (Thermo Fisher Scientific), respectively. Single cardiomyocytes were measured using an image quantitative digital analysis system (NIH Image version 1.6) as described previously (43). The outlines of at least 200 cardiomyocytes were traced in each section.

### Determination of protein and DNA concentrations

The protein levels were determined by DC^TM^ Protein Assay (Bio-Rad). Genomic DNA was extracted from cultured cardiomyocytes using a Hiyield Genomic DNA Isolation Kit (cultured cells) (Cedarlane Laboratories Ltd., Burlington, Canada) and measured by a Nanodrop spectrophotometer (*A*_260_).

### Determination of oxidative stress levels in hearts

The formation of ROS in heart tissue lysates was measured using the Amplex® Red hydrogen peroxide/peroxidase assay kit (Thermo Fisher Scientific), according to the manufacturer's instructions. Briefly, frozen heart tissues were homogenized in an assay buffer. The homogenates (50 μg of protein) were incubated with a fluorescent probe Amplex® Red and hydrogen peroxide/peroxidase at 37 °C. The fluorescent product formed was quantified using a spectrofluorometer measured at 485/525 nm. Changes in fluorescence were expressed as arbitrary units.

Protein carbonyl content was determined using a protein carbonyl colorimetric assay kit (Cayman Chemical Co.) following the manufacturer's instructions. A total of 700 μg of protein was used for each sample.

Lipid peroxidation in heart tissue lysates was assessed by measuring MDA production using a TBARS assay kit (Cayman Chemical) following the manufacturer's instructions. A total of 500 μg of protein was used for each sample.

### Neonatal mouse cardiomyocyte cultures and simulated microgravity

Neonatal mice (born within 24 h) were euthanized by decapitation. Neonatal cardiomyocytes were prepared and cultured in Dulbecco's modified Eagle's medium with 10% newborn calf serum and penicillin-streptomycin according to methods described previously (44).

Right after isolation, cardiomyocytes (1.5 × 10^6^) were cultured with Cytodex 3 microcarrier beads (175-μm particle size, spherical) for 24 h. To simulate microgravity, we dispersed cardiomyocytes on Cytodex 3 microcarrier beads in 5 ml of Dulbecco's modified Eagle's medium with 10% newborn calf serum and penicillin-streptomycin and then inoculated them through the syringe port inside the 5-ml high-aspect ratio vessels of a rotary cell culture system (RCCS-4, Synthecon Inc.). After being assembled, they were placed on their rotary base and maintained in a 37 °C incubator with a 5% CO_2_/air mixture and saturating humidity. Vessel rotation was set at 18 rpm according to our recent report (30). After 24 h, cells were collected for Western blotting analysis. For the treatment with various inhibitors (PD98059 (10 μm), SB23580 (10 μm), and calpain inhibitor-III (10 μm)), the inhibitors or vehicle were added to culture media at the time of inoculation into the rotary device. The cells were incubated under simulated microgravity for 24 h.

### Western blotting analysis

40 μg of protein from heart tissue or cell lysates or 10 μg of protein from isolated membrane lysates were loaded onto SDS-polyacrylamide gels. After electrophoresis, the separate proteins were transferred onto Bio-Rad polyvinylidene difluoride membranes. After blocking in 5% nonfat milk for 1 h, the membranes were incubated with antibodies against NOX4 (1:1000 dilution; Abcam), Rac1 (1:1000 dilution; Abcam), MuRF1 and atrogin-1 (1:1000 dilution; Abcam), Na^+^/K^+^-ATPase (1:1000 dilution; Abcam), p67*^phox^* (1:1000 dilution; Abcam), phosphorylation of Ser-345 and Ser-370 on p47*^phox^* and total p47*^phox^* (1:1000 dilution; Thermo Fisher Scientific Inc.), phosphorylated p38 and total p38 (1:1000 dilution; Cell Signaling Technology), phosphorylated ERK1/2 and total ERK1/2 (1:1000 dilution; Cell Signaling Technology), and GAPDH (1:5000 dilution; Cell Signaling Technology), respectively, followed by secondary relevant antibodies conjugated with horseradish peroxidase. The signals were then developed using an enhanced version of the chemiluminescence reaction.

The protein ladders were purchased from FroggaBio Inc. (Concord, Canada) for cultured cardiomyocytes (245, 180, 135, 100, 75, 63, 48, 35, 25, 20, 17, and 11 kDa) and Thermo Fisher Scientific China Co. Ltd. for heart tissue samples (170, 130, 100, 70, 55, 40, 35, 25, 15, and 10 kDa).

### Quantification of mitochondrial DNA copies by real-time PCR

Total DNA was extracted using a DNA extraction kit (Qiagen), following the manufacturer's instructions. Real-time PCR was conducted using primers specific to the mitochondrial *Mtnd1* region of the mitochondrial genome and β_2_-microglobulin (*B2M*) as a nuclear gene reference. The sequences of primers are as follows: *Mtnd1*, 5′-GAGGGAACCAAACTGAACGC-3′ and 5′-TGGATCCGTTCGTAGTTGGAG-3′; *B2M*, 5′-CAGACTCTGCGATGTTTCCA-3′ and 5′-GCCTGAGCACTTCCAGAAAC-3′. The mitochondrial DNA copies were expressed as the ratio of *Mtnd1* to *B2M*.

### Calpain activity

Calpain activities were measured in tissue and cell lysates (15 μg of protein) using a fluorescence substrate, *N*-succinyl-LLVY-7-amido-4-methylcoumarin (Cedarlane Laboratories) as described previously (35).

### NADPH oxidase activation

NADPH oxidase activation was determined by measuring the translocation of Rac1, p47*^phox^*, and p67*^phox^* to cell membranes. Briefly, cell membranes were isolated from heart tissues using a commercial kit (Beyotime Biotechnology, Shanghai, China) according to the manufacturer's instructions. The protein levels of Rac1, p47*^phox^*, and p67*^phox^* in cell membranes and whole-heart lysates were analyzed using Western blotting. Na^+^/K^+^-ATPase and GAPDH were used as loading controls for the membranes and whole lysates, respectively.

### Xanthine oxidase activity

Xanthine oxidase activity was determined in heart tissue lysates using a commercial assay kit (Beyotime Biotechnology), following the manufacturer's instructions. A total of 100 μg of protein was used for each sample in the assay.

### SOD activity, catalase activity, and GPx activity

These activities were measured in heart tissue lysates using commercial assay kits (Beyotime Biotechnology), following the manufacturer's instructions. A total of 100 μg of protein was used for each sample in the assay.

### Statistical analysis

All data are expressed as means ± S.D. Differences between two groups were compared by unpaired Student's *t* test. For multigroup comparisons, ANOVA followed by Newman–Keuls test was performed. A value of *p* < 0.05 was considered statistically significant.

## Data availability

All data presented are available upon request from Tianqing Peng (tpeng2@uwo.ca or tqpeng@suda.edu.cn).
