# Supplementary material for: Calpain activation mediates microgravity-induced myocardial abnormalities in mice via p38 and ERK1/2 MAPK pathways
Source: J Biol Chem. 2021 Jan 13;295(49):16840–51. doi: 10.1074/jbc.RA119.011890 (PMC7864076; doi:10.1074/jbc.RA119.011890)
Supplement: Supplementary file 1 [file mmc1.pdf]

Supplementary Figure 1

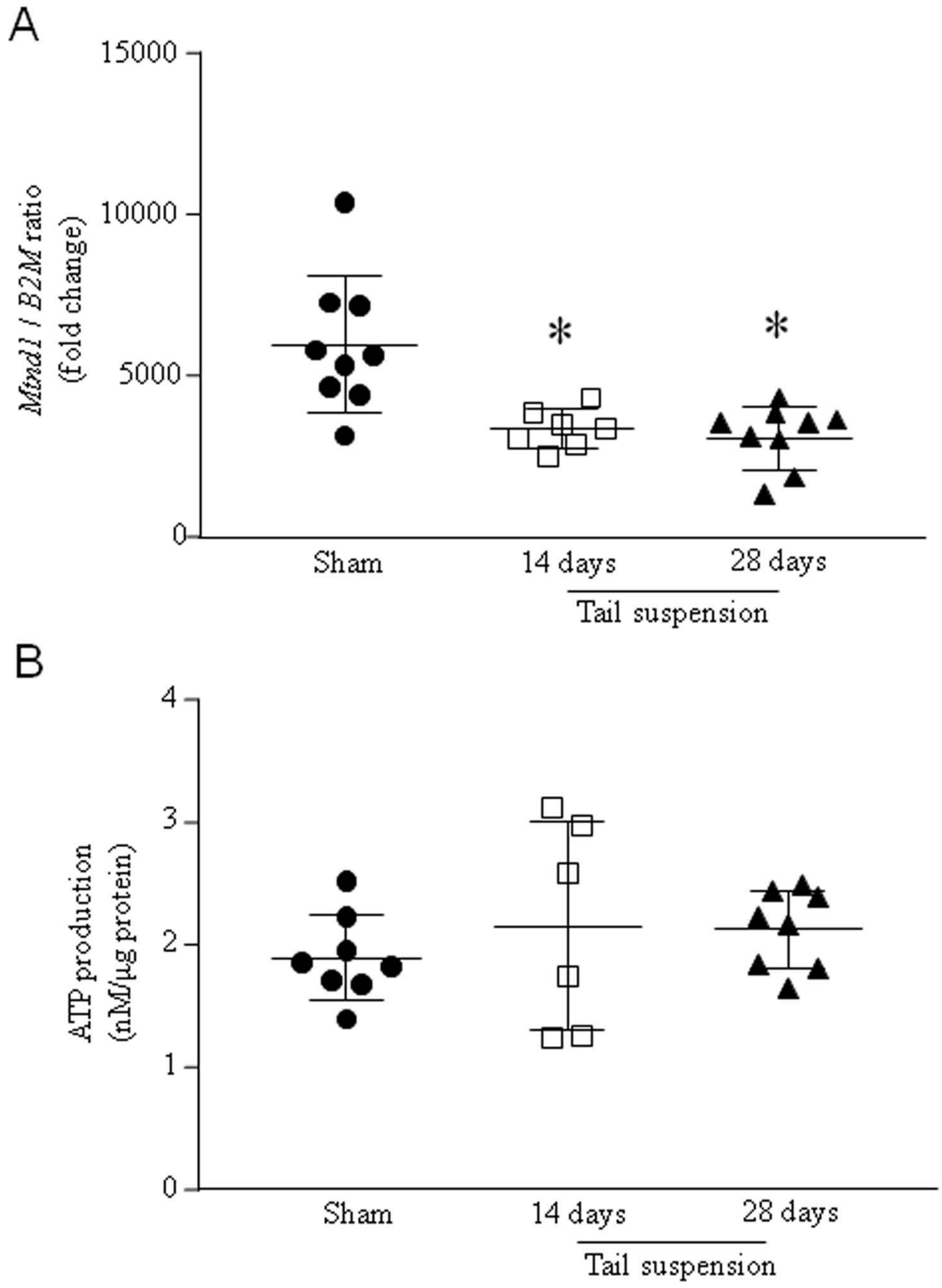

Supplementary Figure 1. Effect of tail-suspension on mitochondrial DNA copies and ATP production in heart tissues. Adult male mice were subjected to tail-suspension for 14 or 28 days. (A) Real-time PCR was conducted to determine the mRNA levels of mitochondrial NADH dehydrogenase subunit 1 (*Mtnd1*) and β2 microglobulin (*B2M*) as a nuclear gene reference. (B) ATP production was measured in heart tissues. Data are mean ± SD, n = 7 - 9. One-way ANOVA followed by Newman-Keuls test was performed for statistical analysis ($F = 10.36$, $P = 0.0007$). *$P < 0.05$ vs. Sham.

Supplementary Figure 2

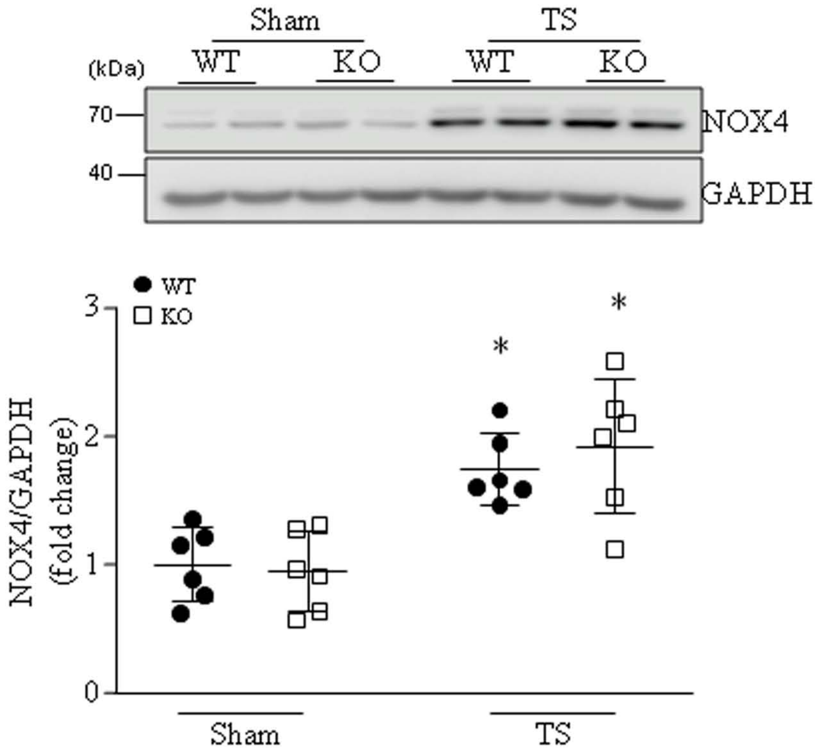

Supplementary Figure 2. Tail-suspension equally results in higher protein levels of NOX4 in wild-type and *Capns1*-knockout mice. Mice with cardiomyocyte-specific deletion of *Capns1* (KO) and their wild-type littermates (WT) were subjected to tail-suspension (TS) for 28 days. Upper panel: a representative western blot from 2 out of 6 different heart tissues for NOX4 and GAPDH. Lower panel: quantitation for NOX4 relative to GAPDH. Data are mean ± SD, n = 6 in each group. Two-way ANOVA followed by Newman-Keuls test was performed for statistical analysis. Interaction: F = 0.6369, P = 0.4342; Row Factor: F = 33.65, P < 0.0001; Column Factor: F = 0.1658, P = 0.6882. **P* < 0.05 vs. Sham + WT or Sham + KO.

Supplementary Figure 3

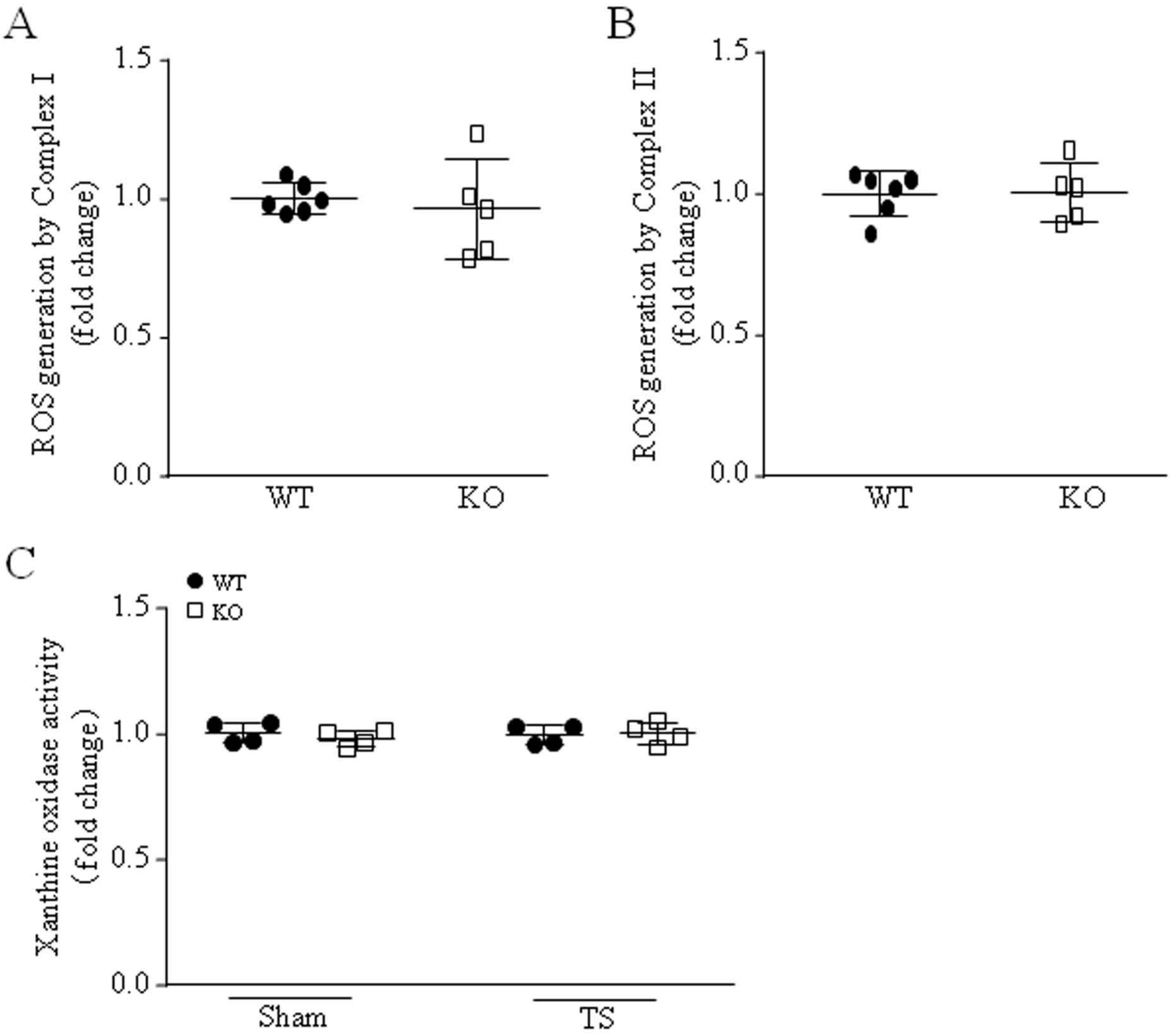

Supplementary Figure 3. Determination of mitochondrial ROS generation in mitochondria and xanthine oxidase activity in heart tissues. Mice with cardiomyocyte-specific deletion of *Capns1* (KO) and their wild-type littermates (WT) were subjected to tail-suspension (TS) for 28 days. (A and B) Mitochondrial ROS generation using complex I (A) and III substrates (B). Unpaired t test was performed for statistical analysis. (C) Xanthine oxidase activity. Data are mean ± SD, n = 4-6 in each group. Two-way ANOVA followed by Newman-Keuls test was performed for statistical analysis. Interaction: F = 0.6026, P = 0.4526; Row Factor: F = 0.06058, P = 0.8097; Column Factor: F = 0.1294, P = 0.7253.

Supplementary Figure 4

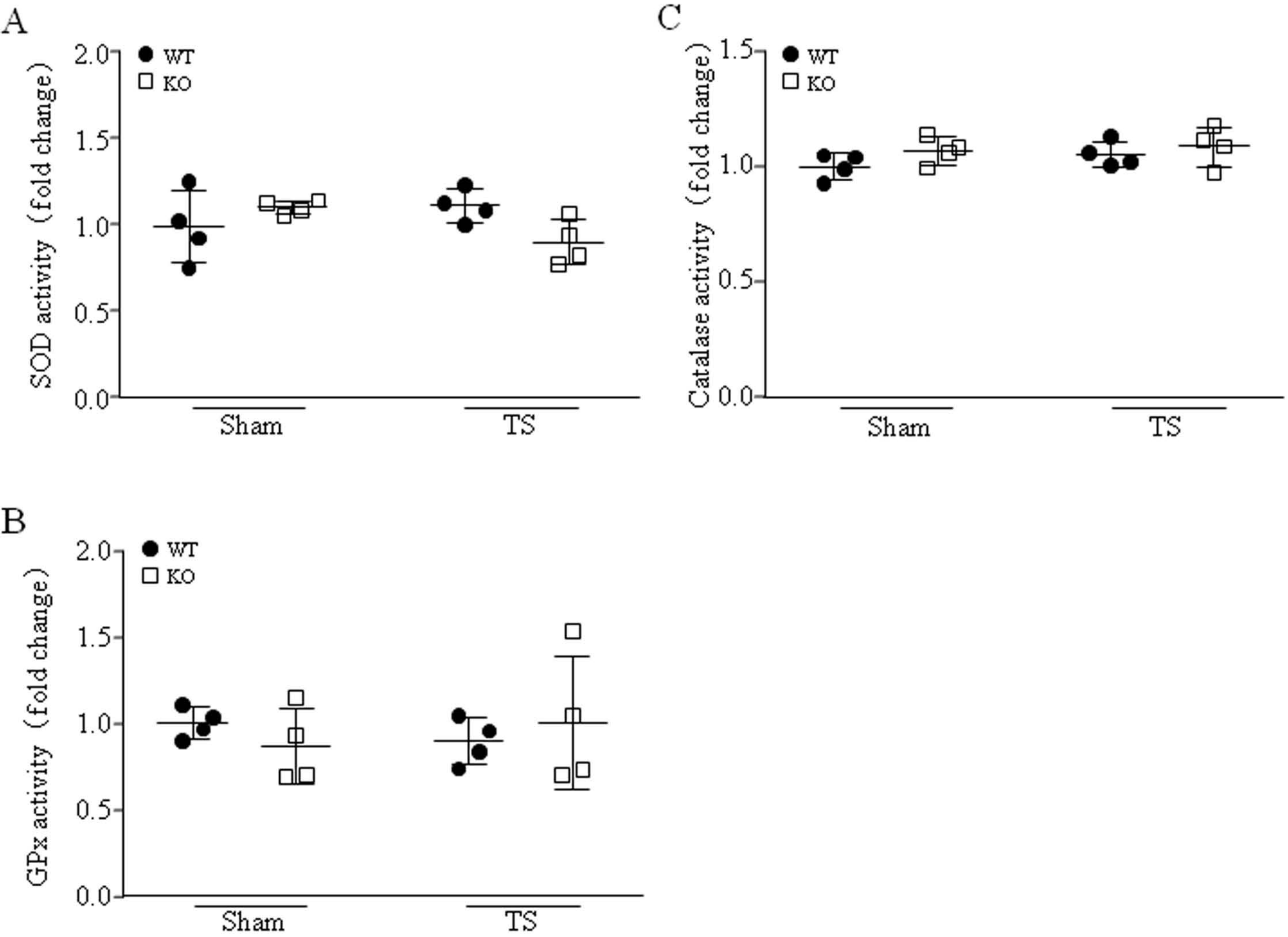

Supplementary Figure 4. Deletion of *Capns1* does not affect tail-suspension-induced SOD activity, GPx activity and catalase activity in mouse hearts. Mice with cardiomyocyte-specific deletion of *Capns1* (KO) and their wild-type littermates (WT) were subjected to tail-suspension (TS) for 28 days. (A) SOD activity, (B) GPx activity and (C) catalase activity were determined in mouse hearts. Data are mean ± SD, n = 4 in each group. Two-Way ANOVA was performed for statistical analysis. (A) Interaction: F = 5.84, P = 0.0325; Row Factor: F = 0.3372, P = 0.5722; Column: F = 0.5337, P = 0.4791. (B) Interaction: F = 1.062, P = 0.3230; Row Factor: F = 0.01619, P = 0.9009; Column: F = 0.0117, P = 0.9157. (C) Interaction: F = 0.2693, P = 0.6132; Row Factor: F = 1.232, P = 0.2888; Column: F = 2.307, P = 0.1547.

Supplementary Figure 5

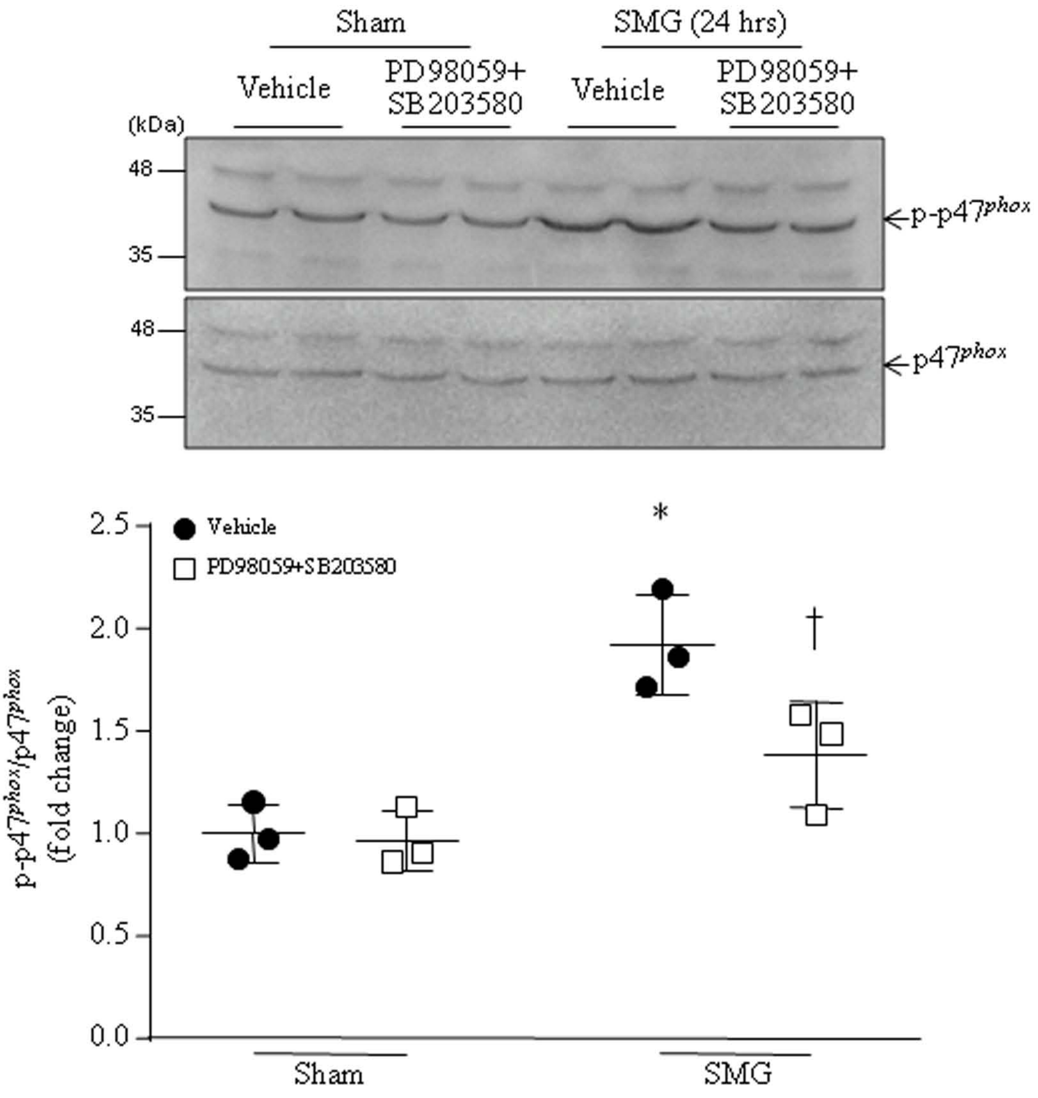

Supplementary Figure 5. Effect of PD98059 plus SB203580 on p47^{phox} phosphorylation. Neonatal mouse cardiomyocytes were subjected to simulated microgravity (SMG) in the presence of PD98059 plus SB203580 (10 µM for each) or vehicle for 24 hrs. Upper panel: A representative western from 3 different cell cultures with each in duplications for phosphorylated p47^{phox} (p-p47^{phox}) and total p47^{phox}; Lower panel: Quantitation of p-p47^{phox} / total p47^{phox} ratio. Data are mean ± SD, n = 3 different cultures. Two-way ANOVA followed by Newman-Keuls test was performed for statistical analysis. Interaction: F = 4.54, P = 0.0657; Row Factor: F = 32.5, P = 0.0005; Column: F = 5.951, P = 0.0406. *P < 0.05 vs. Sham + vehicle and † P < 0.05 vs. SMG + vehicle.

Supplementary Figure 6

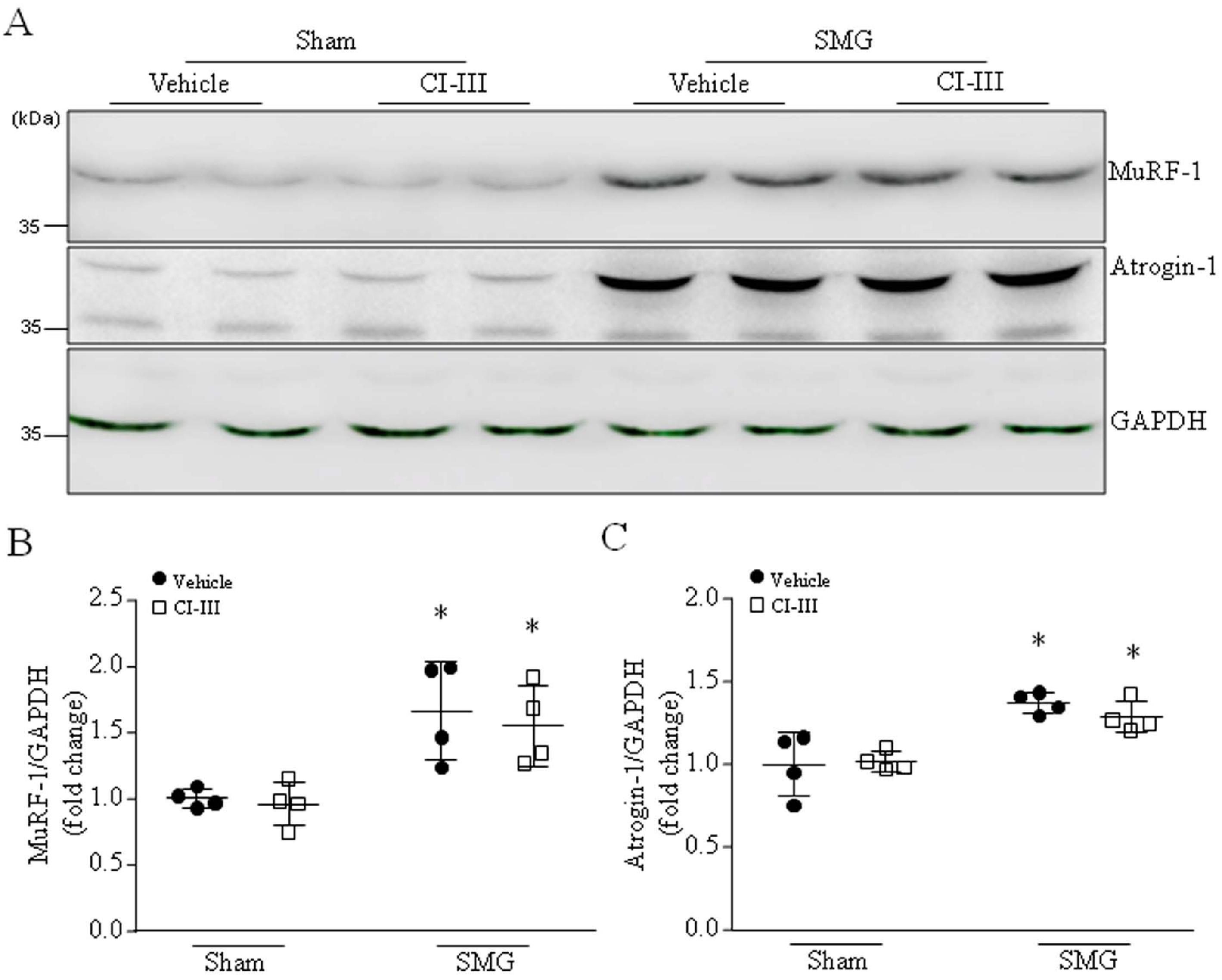

Supplementary Figure 6. MuRF-1 and atrogin-1 expression in cultured cardiomyocytes in response to simulated microgravity. Neonatal mouse cardiomyocytes were subjected to simulated microgravity (SMG) in the presence of calpain inhibitor-III (CI-III) or vehicle for 24 hrs. (A) A representative western from 3 different cell cultures with each in duplications for MuRF-1 and atrogin-1. (B) Quantitation of MuRF-1 protein levels relative to GAPDH. Data are mean ± SD, n = 4 different cultures. Two-way ANOVA followed by Newman-Keuls test was performed for statistical analysis. Interaction: F = 0.07388, P = 0.7904; Row Factor: F = 23.7, P = 0.0004; Column: F = 0.3444, P = 0.5682. *P < 0.05 vs. Sham + vehicle or Sham + CI-III. (C) Quantitation of atrogin-1 protein levels relative to GAPDH. Data are mean ± SD, n = 4 different cultures. Two-way ANOVA followed by Newman-Keuls test was performed for statistical analysis. Interaction: F = 0.782, P = 0.3939; Row Factor: F = 30.48, P = 0.0001; Column: F = 0.3388, P = 0.5713. *P < 0.05 vs. Sham + vehicle or Sham + CI-III.

Supplementary Figure 7

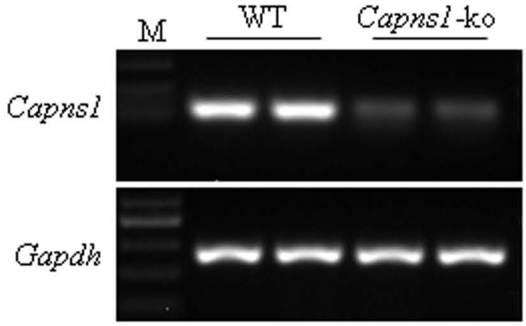

Supplementary Figure 7. *Capns1* mRNA expression in hearts of *Capns1*-ko mice and their wild-type littermates (WT). The mRNA expression of *Capns1* was determined in heart tissues of adult *Capns1*-ko mice and their wild-type littermates by RT-PCR. The representative agarose gel for *Capns1* and *Gapdh* mRNA from 2 different hearts in each group shows that the mRNA levels of *Capns1* are much lower in *Capns1*-ko compared with wild-type mouse hearts. M: DNA marker.

Supplementary Tables

Supplementary Table 1. Body weight, heart weight and lindlimb weight

Group	N	BW (g)	HW (mg)	HLW (mg)	TL (mm)	HW/TL (mg/mm)	HLW/TL (mg/mm)
WT	5	28.25 ± 1.23	115.84 ± 10.39	774.25 ± 33.68	18.52 ± 0.53	6.27 ± 0.67	41.84 ± 2.32
KO	5	27.99 ± 0.61	109.36 ± 7.66	806.37 ± 36.03	18.15 ± 0.33	6.03 ± 0.46	44.44 ± 1.85
TS-WT	7	23.98 ± 1.47 *	86.29 ± 3.21*	487.51 ± 66.60*	18.28 ± 0.82	4.72 ± 0.15*	26.72 ± 3.86*
TS-KO	7	24.60 ± 0.92	100.61 ± 9.78†	497.54 ± 32.92	18.08 ± 0.46	5.57 ± 0.61†	27.53 ± 1.87

WT, wild type; KO, *Capns1* knockout; TS, tail suspension; N, number; BW, bodyweight; HW, heartweight, HLW, hindlimb weight; TL, tibia length. Data are mean ± SD. * $P < 0.05$ vs. WT; † $P < 0.05$ vs. TS-WT.

Supplementary Table 2. Echocardiographic analysis

Group	N	HR (bpm)	LVID;d (mm)	LVID;s (mm)	E/A	EF%	FS%
WT	7	409.86 ± 7.97	3.60 ± 0.11	2.03 ± 0.19	2.08 ± 0.27	75.63 ± 5.02	52.92 ± 6.13
KO	7	441.14 ± 12.26	3.70 ± 0.42	2.17 ± 0.51	1.87 ± 0.13	72.60 ± 9.69	49.58 ± 4.22
TS-WT	7	406.86 ± 25.56	3.88 ± 0.39	2.25 ± 0.67	1.26 ± 0.17*	53.63 ± 10.04*	27.32 ± 6.06*
TS-KO	7	400.71 ± 23.05	3.33 ± 0.33	2.04 ± 0.52	1.48 ± 0.19†	68.58 ± 15.46†	44.44 ± 6.46†

WT, wild type; KO, *Capns1* knockout; TS, tail suspension; N, number; HR, heart rate; LVID;d, left ventricle end diastolic inner diameter, LVID;s, left ventricle systolic inner diameter; E/A, the rate of E over A; EF, ejection fraction; FS, fractional shortening. Data are mean ± SD. * $P < 0.05$ vs. WT; † $P < 0.05$ vs. TS-WT.
